# A novel missense mutation in the *MYH7* gene causes an uncharacteristic phenotype of myosin storage myopathy: a case report

**DOI:** 10.1186/s12881-019-0804-0

**Published:** 2019-05-08

**Authors:** Jean Mamelona, Louisa Filice, Youcef Oussedik, Nicolas Crapoulet, Rodney J. Ouellette, Alier Marrero

**Affiliations:** 1 Department of Neurology, Dr.-Georges-L.-Dumont University Hospital Center, 330 University Avenue, Moncton, NB E1C 2Z3 Canada; 2 Centre de Formation Médicale du Nouveau-Brunswick, 100 Des Aboiteaux Street, Moncton, NB E1A 7R1 Canada; 3 Department of Pathology, Dr.-Georges-L.-Dumont University Hospital Center, 330 University Avenue, Moncton, NB E1C 2Z3 Canada; 4 Molecular Genetics, Dr.-Alfred-Bastarche Laboratory, 37 Providence Street, Moncton, NB E1C 8X3 Canada

**Keywords:** Slow/cardiac beta-myosin heavy chain, Myosin storage myopathy, *MYH7*, Missense mutation, Case report

## Abstract

**Background:**

Few manuscripts have reported phenotypes of skeletal muscle myopathies caused by mutations in the head region of slow/cardiac beta-myosin heavy chain (MyHCI). Among the patients, some of them showed the phenotype of skeletal muscle weakness with the obvious clinical features of cardiomyopathy while others showed pure skeletal muscle weakness with no symptoms of cardiac involvement. Genotype-phenotype relationship regarding the effect of a mutation on MyHCI is complex. Questions regarding why some mutations cause cardiomyopathy or skeletal muscle disorders alone or a combination of both still need to be answered. More findings in genetic variation are needed to extend knowledge of mutations in the *MYH7* gene linked to skeletal muscle disorders.

**Case presentation:**

Here we present a female adult patient with a phenotype of childhood onset of muscular disorders and predominant involvement of thigh muscles with biopsy showing intrasarcoplasmic inclusion bodies. Whole exome sequencing showed that variant c.1370 T > G (p.Ile457Arg) in the *MYH7* gene is a missense mutation possibly linked to the clinical findings. Our patient likely shows an uncharacteristic myosin storage myopathy associated with respiratory and cardiac involvement linked to a missense mutation in the head of MyHCI.

**Conclusions:**

Given this mutation is located within the motor domain of MyHCI, this might affect the regulation of myosin mechano-chemical activity during the contractile cycle. Consequently, this potentially damaging effect can be easily amplified within the network of ~ 300-myosin molecules forming the thick filament and therefore become cumulatively deleterious, affecting, in turn, the overall organization and performance of sarcomere.

**Electronic supplementary material:**

The online version of this article (10.1186/s12881-019-0804-0) contains supplementary material, which is available to authorized users.

## Background

Slow/cardiac beta-myosin heavy chain (MyHCI) is a class of MyHC predominately expressed in the human ventricle and slow-twitch type-I skeletal muscles. It is encoded by the *MYH7* gene and formed by 1935 amino acids (aa) with the head made by aa in the position 1–847 and the rod by aa 848–1935 [1–3]. Mutations involving the secondary structure of amino acids in rod region led predominantly to cardiac phenotypes with a certain number of them resulting in skeletal muscle involvements including Laing distal myopathy (OMIM 160500), Multiminicore disease (OMIM 255320) and Myosin storage myopathy autosomal recessive (OMIM 255160) and autosomal dominant (OMIM 608358). In almost the same way, mutations in the head region result mainly in cardiomyopathies (HCM and DCM), very few of them leading to skeletal muscle myopathies [3, 4].

To the best of our knowledge, 5 manuscripts have so far reported phenotypes of skeletal muscle myopathies caused by mutations in the head region of MyHCI. They were observed in 4 female patients aged from 38 to 53, all with adult onset, and in 1 child of 7-year-old [5–8]. Among them, 3 patients showed the phenotype of skeletal muscle weakness along with obvious signs of cardiac muscle issues [5–7] while 2 of them showed no signs of cardiac involvement [8]. Even though these mutations have been linked to the phenotypes mentioned above, their full and comprehensive characterization is yet to come to make a direct association between mutations in this specific area of the head of MyHCI and myopathy of skeletal and cardiac muscles. Questions regarding why some mutations cause cardiomyopathy or skeletal muscle disorders alone or a combination of both still need to be answered. More findings in genetic variation would extend knowledge of mutations in the *MYH7* gene linked to skeletal muscle disorders.

## Case presentation

**Fig. 1:**
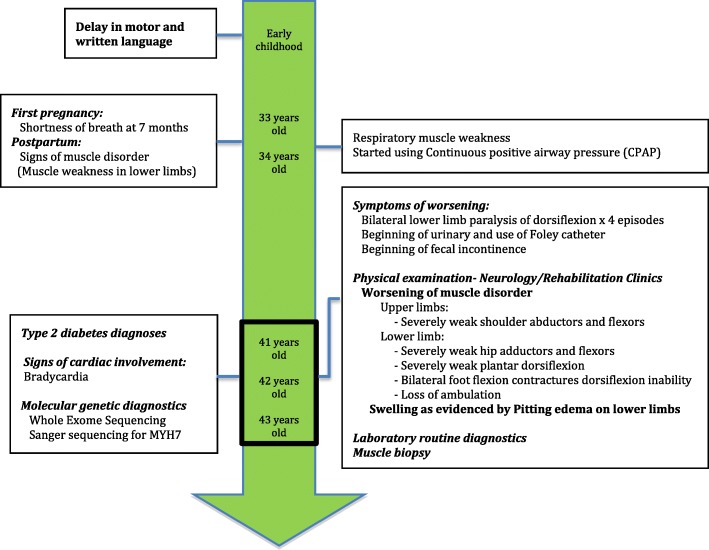
Relevant history of the patient organized into a timeline

A 41-year-old non-smoker obese female patient was examined at the Department of Neurology and the Center for rehabilitation. Relevant history of the patient is summarized on a timeline in Fig. 1. She showed an onset of neuromuscular disorders during early childhood with a delay in motor and written language development. She had a hard time to complete physical exercise at school and always finished last. There was nothing really alarming in terms of muscle disorder until her first pregnancy at the age of 33 during which she experienced shortness of breath (dyspnea) at the 7 months of gestation and showed signs of weakness of lower limbs afterwards. At the age of 34 she showed worsening of breath symptoms, suffered from sleep apnea and started using a mechanical ventilation machine. Alarming symptoms of skeletal muscle disorders occurred immediately after delivery with progressive but rapidly incapacitating weakness of lower limbs. This worsened during the 3 following years, a period during which she first started being unable to get up by herself and then was unable to climb stairs. At the age of 37, she started to use a cane, then a walker for her daily walking needs. At the age of 39, she started using a wheelchair to move outdoors. Despite these signs of progressive muscle weakness, she never had a thorough neuromuscular investigation. It was wrongly thought that her health problems, in general, was mainly related to being overweight. At the age of 41, she had four episodes of lower limb paralysis during which she was completely unable to move her legs and support her weight. She did not seek medical consultation for the first three episodes. For the fourth, she was admitted to the emergency room (ER) and first referred to the Department of Neurology and then to the Center for Rehabilitation.

The family history showed that her mother died at the age of 66 from a heart attack associated with non-compaction cardiomyopathy. There is nothing remarkable in terms of muscle disorders in her father, brother, and sister. However, her only daughter, now at the age of 11, shows signs of muscle disorders with congenital muscular torticollis, excessive growing pains as well as underdeveloped muscles in half of the body.

Physical examinations conducted following her admission to the ER at the age of 41 showed normal tone/bulk of the arm muscles. However, muscles in shoulders and upper and lower limbs showed bilateral weakness. Deltoids, biceps and triceps showed moderate weakness with MRC scale of 3/5. Fine finger movements were intact. There was no pronator drift. In the lower limbs, all muscles examined showed the same severity of weakness (2/5) except quadriceps which showed mild weakness (4/5). Hip flexors were extremely weak (1/5); hip abductors and adductors were mildly weak (4/5). Knee extensors and flexors were moderately weak (3/5). Dorsiflexion of feet was severely weak (2/5). Plantar flexors were severely weak (2/5). Deep tendon reflexes were 2+ in the arms, absent in the patella and Achilles. The toes were down-going. No sensory deficit was observed. No sign of dysphagia or involvement of ocular muscles was observed.

Additional physical examination conducted during the 2-year follow-up (at the age of 43) showed no worsening of muscle strength. However, this brought new information about the weakness of other muscles, notably in the shoulders, with extremely weak abductors and flexors (1/5). The patient showed decreased perception of vibration in the lower limbs. She reported that she experienced occasional dysphagia.

Electrophysiological exams showed normal nerve conduction velocities for upper and lower limbs, with all SNAP and CMAP amplitudes in the range of normal values. However, needle EMG revealed a tendency of myotonic potentials, generated by needle insertion. Right deltoids, biceps and ulnar-innerved first dorsal interosseus muscles showed myotonic potentials as well. The right tibialis anterior showed 2+ polyphasic motor unit potentials with myotonic potentials. The right medial gastrocnemius, vastus medialis and vastus lateralis showed myotonic tendencies as well.

**Fig. 2:**
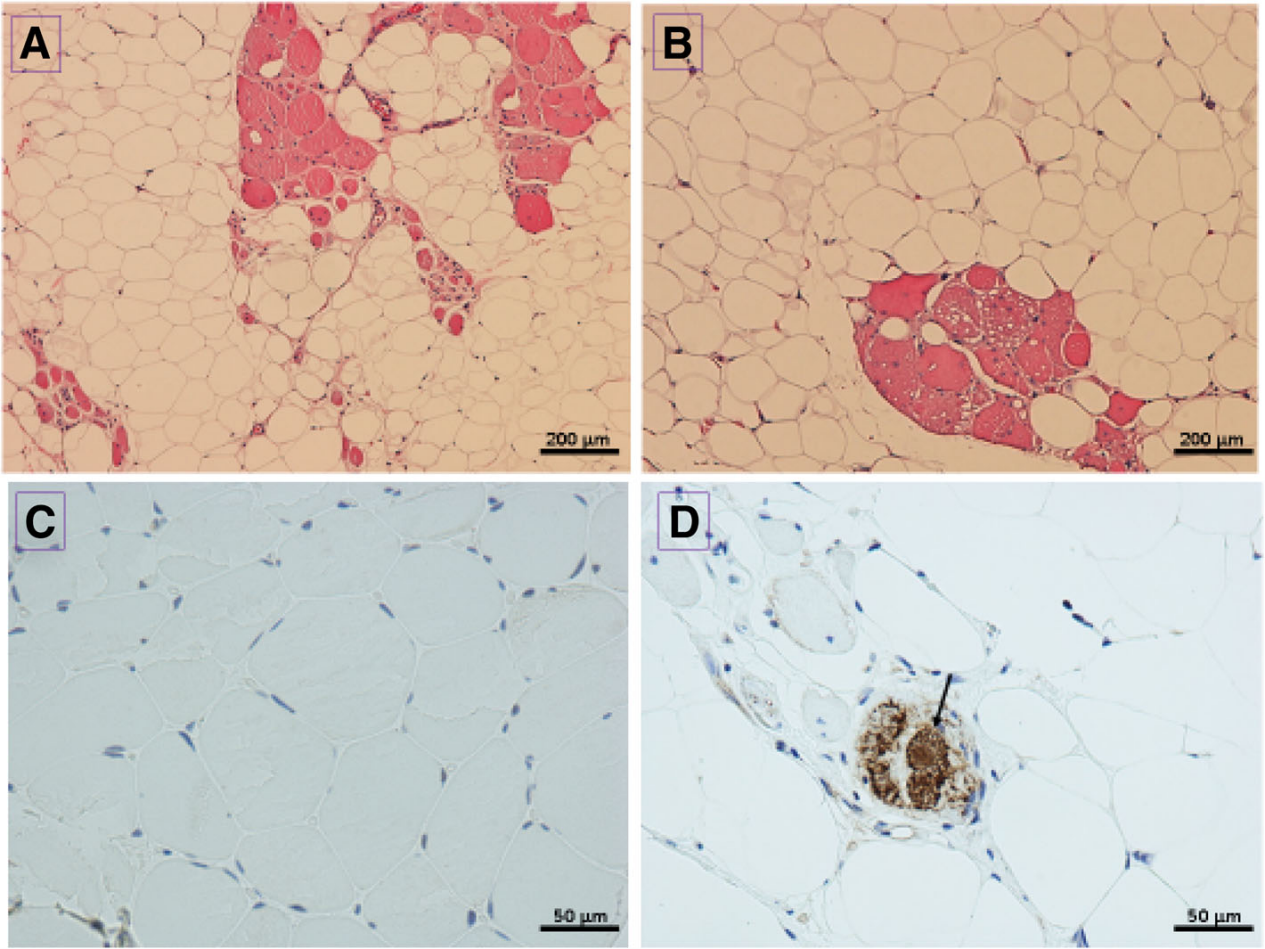
Results of muscle biopsy of left quadriceps. **a** H&E stain showing end-stage muscle. Scattered clusters of viable muscle fibres show marked variation in fiber size and numerous internal nuclei. **b** H&E stain showing scattered fibers with abundant intrasarcoplasmic vacuoles. **c** Negative control for p62 stain. **d** Transverse view of fibres displaying dark coloured intrasarcoplasmic inclusion bodies on p62 stain (Black arrow). Original magnifications: A: 10×, B: 10×, C: 40×, D: 40×

Examination of muscle biopsy of left quadriceps showed features of an end-stage process, consistent with a severe, chronic myopathy. There are scattered clusters of viable muscle fibers which showed myopathic features in the form of a marked variation in fiber size and numerous internal nuclei. In addition, there are scattered fibers with abundant intrasarcoplasmic vacuoles (Fig. 2a, b). Given we could not clearly observe the presence of hyaline bodies on H&E staining we decided to proceed with p62 immunostaining, a well-known technique for revealing the presence of inclusion bodies [9]. Immuno-histochemical detection of p62 was performed on a Leica Bond III automated stainer. Following digestion in a low pH citrate solution, sections were incubated in a primary mouse monoclonal anti-p62 antibody, diluted 1:50 (BD Transduction Laboratories Catalog Number 610833). Detection of bound antibody was achieved using the Leica Bond Polymer Refine Detection kit, comprising the secondary antibody, the substrate chromogen DAB (3,3′-Diaminobenzidine tetrahydrochloride hydrate) and the Hematoxylin counterstaining solutions. Immunostaining for p62 revealed, in a proportion of the surviving muscle fibers, diffusely distributed, small intermyofibrillar dots or, more commonly, larger central or eccentric sarcoplasmic inclusion bodies (Fig. 2c, d). Examination of heart function using Holter ECG monitor carried out following her admission to the ER showed no clinically significant implication of cardiac involvement. However, the patient complained about repeated episodes of oppressive chest pain during the following years. Additional examination conducted during the 2-year follow-up showed signs of cardiac involvement with bradycardia of 58 beats per minute.

Regarding respiratory involvement, the patient suffered from dyspnea since the age of 34. Examination of lung function conducted following her admission to ER and during the follow-ups showed worsening of her respiratory condition. Spirometry test conducted at the age of 44 showed a very weak pulmonary function, with forced vital capacity (FVC) of 27% and forced expiratory volume in a second (FEV1) of 29% of normal values.

The blood samples were collected for whole-exome sequencing (WES) to detect mutations potentially involved in the phenotype of neuromuscular disorders observed for this patient. The genomic DNA was extracted from whole blood and subsequently subjected to whole-exome DNA library construction using the Ion AmpliSeqTM Exome RDY panel (Thermo Fisher Scientific) essentially as described in the manufacturer’s protocol, with barcode incorporation. For the sequencing, samples were loaded on an Ion HI-Q PI Chip v3 and placed onto the Ion Proton instrument (Thermo Fisher Scientific) together with an Ion PI HI-Q sequencing 200 Kit (Thermo Fisher Scientific) and sequenced for 520 cycles according to the manual (See Additional file 1: Table S1 for parameters). All candidate mutations found by WES were validated by direct Sanger sequencing (See Additional file 2: Figure S1 for the filtering process). DNA sequences were obtained from the University of California Santa Cruz (UCSC) Genome Browser. Predesigned primers were directly purchased from Thermo Fisher Scientific (See Additional file 1: Tables S2 for details). Amplicons were sent to Genewiz (https://www.genewiz.com) for Sanger sequencing.

**Fig. 3:**
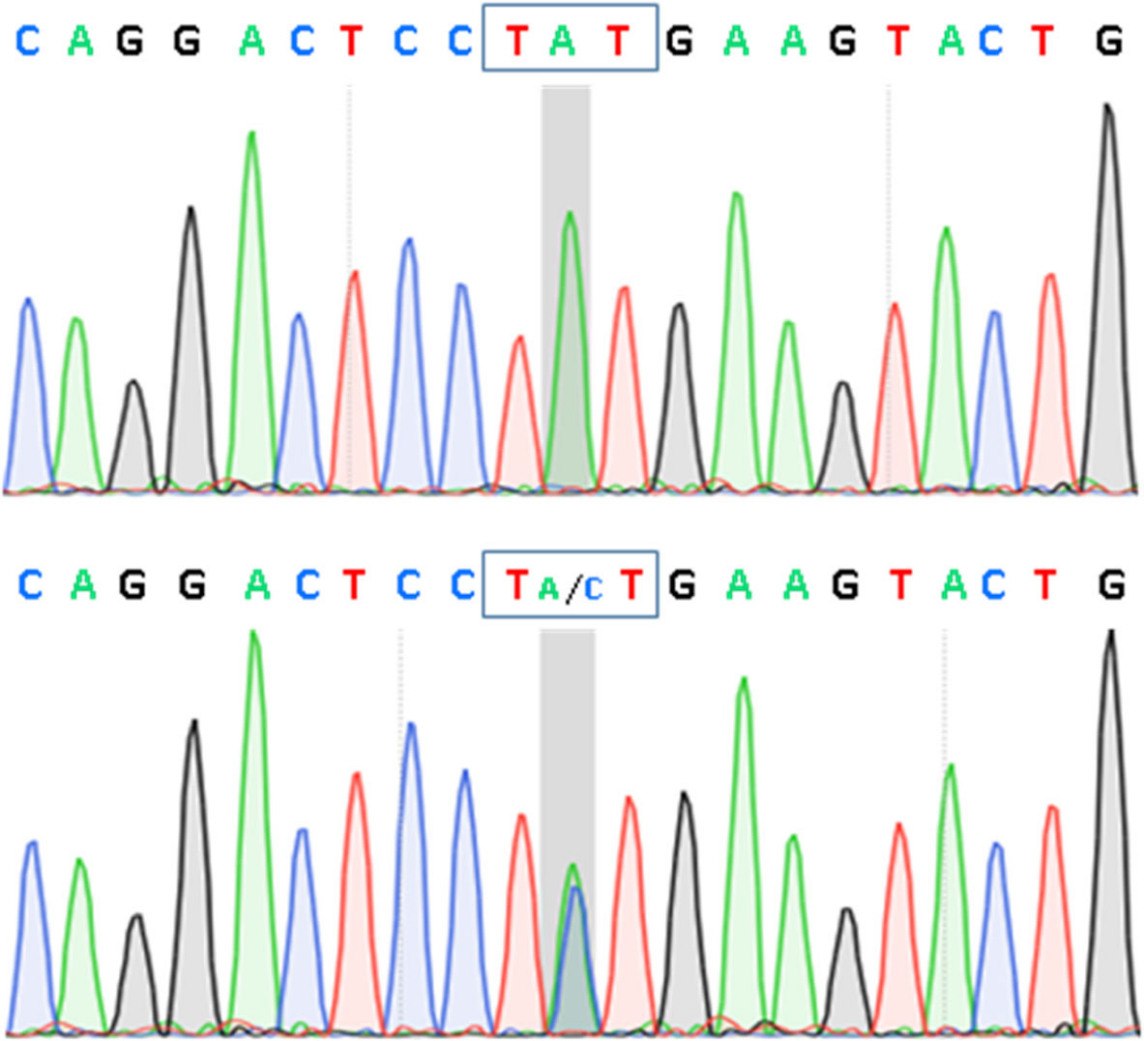
Partial electropherograms of the genomic region covering the *MYH7* gene, with the representation showing the coding strand. Top. Control subject; Bottom. Patient, carrying variant NM_000257.3: c.1370 T > G (p.Ile457Arg) in a heterozygous status. The location of mutated nucleotide is highlighted in grey

Whole exome sequencing showed that a novel variant NM_000257.3: c.1370 T > G (p.Ile457Arg) in the *MYH7* gene is a missense single nucleotide variant possibly linked to the clinical findings, found in the DNA of the patient as heterozygous (Fig. 3). The novel variant has been submitted to ClinVar database; with the assigned accession number SCV000804311. Two other candidate mutations were identified in the DNA of this patient, namely NM_003085.4:c.368C > A (p.Pro123His) in the SNCB gene and NM_001001557.3:c.746C > A (p.Ala249Glu) in the GDF6 gene (Additional file 1: Table S3). However, both mutations were discarded after filtering only genes involved in neuromuscular functions that potentially cause clinical features of muscle myopathy observed in this patient (Additional file 2: Figure S1). Regarding the novel variant NM_000257.3: c.1370 T > G (p.Ile457Arg), bioinformatics analyses showed that nucleotide T coding at the position 1370 of the *MYH7* gene is highly conserved across 44 vertebrate species (PhyloP at 1.76). Amino acid substitution from Isoleucine (I) to Arginine (R) at the position 457 of MyHCI suggested a high impact on protein structure (Grantham at 97). Results of analyses obtained from VarSome (https://varsome.com/variant/hg19/NM_000257.3%3Ac.1370T>G) suggested a classification of Likely pathogenic for this variant, with evidence of Pathogenic computational results coming from 8 various prediction software including DANN, GERP, dbNSFP.FATHMM, MetaLR, MetaSVM, MutationAssessor, MutationTaster and PROVEAN (vs no benign predictions). Also, the results of analyses using the recommendation of the ACMG and the AMP suggested the same classification of Likely pathogenic for this variant, with combined criteria of 2 moderate (PM1 and PM2) and 2 supporting (PP2 and PP3) [10].

## Discussion and conclusions

The novel variant NM_000257.3: c.1370 T > G (p.Ile457Arg) reported in the present study results in a single amino acid change p.Ile457Arg in motor domain of MyHCI, spanning from position 87 to 778. This change would be located toward N-terminal region of the 13th β-strand within this domain, which spans from position 455 to 463 [UniProtKB-P12883 (MYH7_HUMAN); https://www.uniprot.org/uniprot/P12883]. At the molecular level, this brings a positively charged hydrophilic amino acid Arginine, donator of H bond instead of Isoleucine which is neutral and hydrophobic. Accordingly, this change in amino acid sequence potentially impairs electrostatic interaction between amino acid residues and might destabilize the β-strand structure. This corroborates with result of our bioinformatics analysis of damaging impact of this variant on protein structure and the evidence of Pathogenic significance coming from 8 prediction software mentioned above.

In terms of structure/function, the amino acid sequence affected by this mutation would be in the highly conserved central 50-kDa cleft of myosin motor domain [11–14]. Experiments showed that mutations within this cleft might result in myosins that were not fully functional, with defects ranging from ATP nonhydrolyzers to myosins whose enzymatic and mechanical properties are uncoupled [14]. At the muscular contraction unit level, bioinformatic analysis data could provide a better understanding of how this unique mutation could be pathogenic. A network of about 300 myosin molecules forms a single thick filament, with unique structural relationships [4]. Within this well-organized network, the damaging effects on protein structure in the motor domain can easily be magnified and become cumulatively harmful. This could therefore affect the organization and overall performance of sarcomere. Previous study showed that mutations within motor domain modulate myosin activity and lead to myofibril disorganization [15]. Even though segregation analysis is hampered by the lack of DNA test and clinical data in relatives, most likely this variant could be contributory to the phenotype of muscular disorders observed in this patient.

To the best of our knowledge, this current study reports the sixth case of mutations in the *MYH7* gene portion encoding for the head region of MyHCI linked to phenotypes of muscle weaknesses. All the mutations reported so far, results in a change in the primary structure of proteins from one amino to another at different positions within the motor domain [5–8], including the one in the present study. There are variations in the affected muscles and the severity of the myopathy, suggesting that the resulting phenotype depends on the location of the mutation and the amino acid change on the structure of the resulting protein. Unlike the previously reported cases, for this current case, there is a combination of skeletal, respiratory and cardiac muscle involvement (Table 1). Our patient apparently showed childhood onset and in terms of overall clinical features, she shows different characteristic compared to the previously reported cases. Muscle weakness for our patient is more advanced in the most affected parts of the body (1/5 to 2/5) compared to previous cases (3/5 to 4/5). The most affected muscles for her are in lower limbs (2/5) and in the hip (flexors 1/5) while there was no apparent predominance of muscle weakness in previously reported cases [5–8].

**Table 1:** Clinical features of mutations in the head region of MyHCI linked to phenotypes of myopathy

Age Sex	Onset	Further weakness distribution				Additional involvements			Mutation		Ref.
	Age Symptoms	Proximal	Distal	Axial	Lower limbs	Hip	Cardiac	Respiratory	Base	Protein	
58 F	Adult Thigh muscle weakness	ND	ND	Neck flexors	Tibialis anterior (4/5)	Psoas (4/5)	HCM	ND	c.745_746delCGinsGA	p.Arg249Glu	[7]
40F	Adult Tibialis anterior weakness	No	Yes	No	No	No	No	No	c.1322C > T	p.Thr441Met	[8]
07 M	Child (Age of 11 months) Walking on the toes	No	Yes	No	No	Hip flexors	Yes	No	c.1322C > T	p.Thr441Met	[5]
40 F	Child Overall fatigue Lower limb weakness	Deltoids, biceps and triceps (3/5)	Yes	No	Foot dorsiflexion (2/5) Plantar flexors (2/5)	Hip flexors (1/5), abductors and adductors (4/5)	Yes	Yes	c.1370 T > G	p.Ileu457Arg	This study
39F	Adult Hands onset	Yes	Yes	Yes	No	No	No	No	c.1780C > A	p.Leu594Met	[7]
53F	Adult Pain and weakness of forearms and lower legs	Yes	Intrinsic muscles of hands (4/5)	ND	Dorsiflexors of the ankle and great toe (4/5) Bilateral hypertrophy of Tibialis anterior	ND	HCM	ND	c.1816G > A	p.Val606Met	[6]

All positions are based on numbering using transcript NM_000257.3

In this study, we report that the novel missense mutation NM_000257.3:c.1370 T > G (p.Ile457Arg) could be linked to the phenotype observed in our patient. Mutation at the same position has been previously reported in one patient but with different amino acid substitution, i.e. p.Ile457Thr [3, 16]. Authors linked the mutation to a phenotype of hypertrophic cardiomyopathy and did not mention any involvement of skeletal muscle weakness. The discrepancy between the 2 phenotypes observed in patients carrying missense mutation at the same position tends to point out the fact that the genotype-phenotype correlation might depend on the amino acid change on the structure of the resulting protein. Regarding the overall clinical features, our patient apparently showed the onset of muscular disorder during her childhood notably with lower limb fatigue. Presumably, this muscular disorder progressed during the first three decades of her life and consequently, the damages have accumulated. Then, her condition has worsened and became seriously incapacitating in her early forties. This seems to be confirmed by the muscle at the end-stage observed during the biopsy, suggesting that at some level, damage has accumulated in this patient’s muscles. Myopathic features observed for this patient are like those previously observed in adulthood manifestations of myosin storage myopathy including marked variation in fibre size and numerous internal nuclei [17]. In addition, several intrasarcoplasmic inclusion bodies were present on p62 staining. This suggests that our patient might show an indication of a protein aggregation disease [9]. All clinical features taken together, with the mutation in the *MYH7* gene and the presence of intrasarcoplasmic inclusion bodies, tend to point out that this patient seems to be showing overall characteristics of myosin storage myopathy autosomal dominant (MSM; OMIM 608358). But, this would be an uncharacteristic MSM given the fact this is the first case of MSM linked to a mutation within the motor domain of MyHCI. All mutations in the *MYH7* gene linked to MSM are in the rod region of MyHCI [18]. This case expands our knowledge about the phenotypic spectrum of mutations within the head of MyHCI. The results from this study have some limitations and data should be interpreted with caution. As we mentioned above, it was not possible to have DNA test results and clinical data for the parents of the patient. First, it is not clear if the mutation is de novo or hereditary. Second, we have been unable to demonstrate the segregation within the family.

## Additional files

Additional file 1: **Table S1.** Sequencing parameters. **Table S2.** Detailed information for primers used for Sanger sequencing. **Table S3.** Supplementary information on candidate exome variants filtered by database. (DOCX 18 kb)

Additional file 2: **Figure S1.** Filtering process using Genomatix GeneGrid and Qiagen Ingenuity Variant Analysis™ software. (DOCX 42 kb)

## Abbreviations

ACMG: American College of Medical Genetics and Genomics
AMP: Association for Molecular Pathology
CMAP: Compound muscle action potential
ECG: Electrocardiogram
EMG: Electromyography
FEV1: Forced expiratory volume in a second
FVC: Forced vital capacity
H&E: Hematoxylin and Eosin
HCM: Hypertrophic cardiomyopathy
MRC: Medical Research Council
MSM: Myosin Storage Myopathy
MyHC: Myosin heavy chain
MyHCI: Slow/beta-cardiac myosin heavy chain
SNAP: Sensory nerve action potential
