# Supplementary material for: A novel missense mutation in the MYH7 gene causes an uncharacteristic phenotype of myosin storage myopathy: a case report
Source: BMC Med Genet. 2019 May 8;20:78. doi: 10.1186/s12881-019-0804-0 (PMC6507130; doi:10.1186/s12881-019-0804-0)
Supplement: Supplementary file 1 — Table S1. Sequencing parameters. Table S2. Detailed information for primers used for Sanger sequencing. Table S3. Supplementary information on candidate exome variants filtered by database. (DOCX 18 kb) [file 12881_2019_804_MOESM1_ESM.docx]

Table S1. Sequencing parameters

| Parameters | Number |
| --- | --- |
| Mean coverage | 118 x |
| Mean read length | 159 bp |
| Total number of reads | 38,955,799 |
| Total number of bases (AQ20) | 5602.8 Mbp |

Table S2. Detailed information for primers used for Sanger sequencing

| Gene | Accession # | Forward primer | Reverse primer |
| --- | --- | --- | --- |
| GDF6 | Hs00295965 | GACTTCTTGCCGTGCCGCTT | CAGCTCTTCCCTTGCCTTTCG |
| MYH7 | Hs00135440 | GCTGGTCCCCTCCATGTCAA | GGTGAAAGTGGGCAATGAGTAC |
| SNCB | Hs00366501 | CCATCTCATGCCAGGGATGTC | AAGGATGAGGCATAAGCTGGTGA |

Table S3. Supplementary information on candidate exome variants filtered by database

| Chr.Position | Gene | Mutation | Mode, Inheritance | dbSNP ID | Grantham | PhyloP | Significance | Disease,OMIM # |
| --- | --- | --- | --- | --- | --- | --- | --- | --- |
| Chr8:97157413 | GDF6 | c.746C>A p.Ala249Glu | Heterozygous,  AD | rs121909352 | 107.0 | 0.03 | Uncertain Significance | Klippel-Feil syndrome 1  OMIM 118100 |
| Chr14:23898201 | MYH7 | c.1370T>G p.Ile457Arg | Heterozygous, AD | ND | 97.0 | 1.76 | Likely pathogenic | Myosin storage myopathy  OMIM 608358 |
| Chr5:176048219 | SNCB | c.368C>A p.Pro123His | Heterozygous,  AD | rs104893937 | 77.0 | 2.42 | Uncertain Significance | Dementia with Lewy bodies  OMIM 127750 |

Abbreviations: Chr, Chromosome; AR, autosomal recessive; AD, autosomal dominant; NS, non-synonymous; SNV, single nucleotide variants;
ND, Not determined. Chr. positions are established on Assembly GRCh37. Transcript IDs: MHY7 (NM_000257.3); SNCB (NM_003085.4));
GDF6 (NM_000493.3). Significance has been determined using the recommendation of the ACMG and the AMP.
