# Supplementary material for: A novel missense mutation in the MYH7 gene causes an uncharacteristic phenotype of myosin storage myopathy: a case report
Source: BMC Med Genet. 2019 May 8;20:78. doi: 10.1186/s12881-019-0804-0 (PMC6507130; doi:10.1186/s12881-019-0804-0)

Figure S1.

Filtering process using Genomatix GeneGrid and Qiagen Ingenuity Variant Analysis™ software.

AR: autosomal recessive; AD: autosomal dominant; AQ: alignment quality; ExAC: The Exome Aggregation Consortium; HGMD: The Human Gene Mutation Database; gnomAD: The Genome Aggregation Database;
NHLBI ESP: The National Heart, Lung, and Blood Institute Exome Sequencing Project


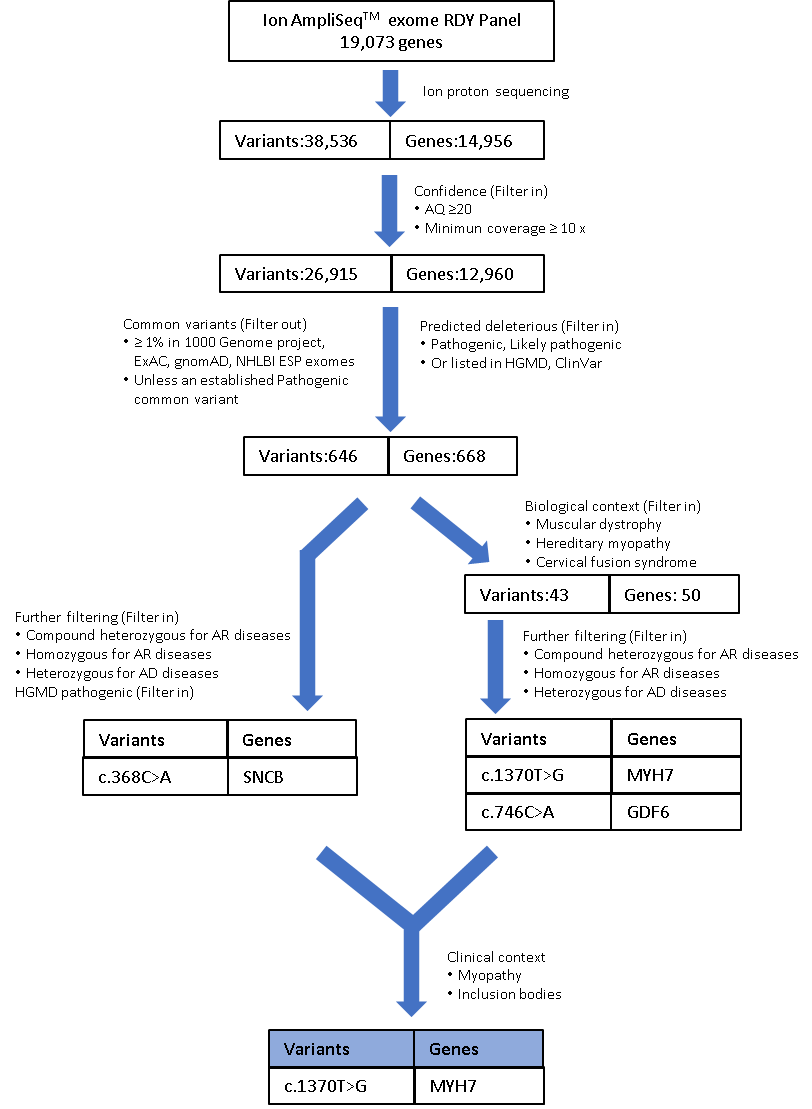

Supplement: Supplementary file 2 — Figure S1. Filtering process using Genomatix GeneGrid and Qiagen Ingenuity Variant Analysis™ software. (DOCX 42 kb) [file 12881_2019_804_MOESM2_ESM.docx]
